# Bilateral ilio-sacral screw fixation of sacroiliac joint using a single guidewire: a technical case report

**DOI:** 10.1097/RC9.0000000000000194

**Published:** 2026-02-05

**Authors:** Nitesh Raj Pandey, Sulabh Kumar Shrestha, Sanjeev Acharya, Bibek Banskota

**Affiliations:** Orthopedic, Pelvi Acetabular and Arthroplasty Unit, Baidya and Banskota Hospital, Lalitpur, Nepal

**Keywords:** bilateral sacroiliac joint disruption, percutaneous sacroiliac screw fixation, single trans-sacral guidewire

## Abstract

**Introduction:**

Bilateral sacroiliac joint (SIJ) disruption is an uncommon, unstable pelvic injury with high morbidity. Percutaneous SI screw fixation is a well-established treatment but typically requires separate guidewire passes, increasing operative time and radiation exposure.

**Case and Technique:**

We report a 22-year-old male with bilateral pubic rami fractures and bilateral SIJ disruptions. After reduction and fixation of anterior ring, a single guidewire was passed from the left to the right SIJ, and bilateral screws were inserted over it under fluoroscopic guidance.

**Results:**

Bilateral fixation was successfully achieved with correct screw placement, effective compression, and intact L5 nerve function. This approach reduced operative time and fluoroscopic exposure.

**Conclusion:**

Single-guidewire bilateral SIJ fixation is a simple, effective technique that decreases operative time and radiation exposure while ensuring safe, stable fixation. Careful fluoroscopic monitoring is essential, and the method is feasible in both obese and nonobese patients.

## Introduction

The global rise in high-energy trauma has led to an increased incidence of unstable pelvic injuries, commonly seen in polytrauma patients and associated with high morbidity and mortality^[1,2]^. Pure sacroiliac joint (SIJ) dislocations are particularly challenging due to complete ligamentous disruption, resulting in pelvic instability, ilio-sacral misalignment, and poor functional outcomes^[3]^. Complications of SIJ injuries include pelvic deformity, persistent pain, functional limitation, as well as trauma-related issues such as hemorrhage, visceral or soft tissue injury, and thromboembolism from prolonged immobility^[4]^.

Percutaneous fixation of pelvic injuries, first introduced by Routt in 1993^[5]^ has become a common technique to supplement open or closed reduction for posterior pelvic ring fractures. This method provides biomechanical stability comparable to open reduction and internal fixation with plates and screws, while offering advantages such as minimal invasiveness, reduced blood loss, and lower infection rates^[6]^. However, percutaneous fixation carries risks including neurological injury (0–8%) and screw malposition (2–12%)^[7,8]^.

**HIGHLIGHTS**

- Minimally invasive: reduces operative time, soft tissue damage, and potential complications.
- Clinical feasibility: demonstrates safe and effective stabilization in a neglected vertical shear pelvis fracture.
- Reproducibility: offers a stepwise method that can be adopted in similar complex pelvic injuries.

Bilateral SIJ disruption are especially challenging, as achieving accurate screw placement with minimal complications and radiation exposure is difficult. This technique proposes placing a single long guidewire across both SI joints to enable bilateral fixation.

### Patient and methods

A 22-year-old male, weighing 68 kg and measuring 5 feet 6 inch in height, presented to the hospital following a road traffic accident. Imaging studies, including plain radiographs and subsequent computed tomography (CT) scans, demonstrated bilateral superior and inferior pubic rami fractures, accompanied by bilateral SIJ disruptions. This work has been reported in line with the SCARE 2025 criteria^[9]^.

### Surgical technique

Following induction of general anesthesia, the patient was positioned supine on a radiolucent operating table, with the body placed near the edge to facilitate optimal C-arm maneuverability and allow for intraoperative fluoroscopic imaging in multiple planes, including inlet, outlet, anteroposterior (AP), iliac oblique, and obturator outlet views.

**Figure 1.**
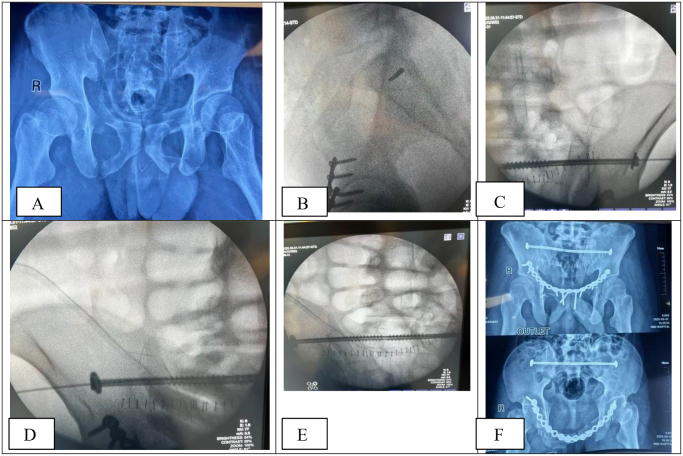
(A) AP pelvic radiograph showing bilateral superior and inferior pubic rami fractures with SIJ disruptions. (B) Lateral fluoroscopic view showing the left-sided guidewire entry point for an S1 ilio-sacral screw. (C, D) A 6.5 mm partially threaded cannulated screw was placed first on the left, then on the right, using the same guidewire. (E) Outlet fluoroscopic image post-fixation showing bilateral ilio-sacral screws placed over a single guidewire. (F) Inlet and outlet view post-fixation demonstrating a well-stabilized pelvic ring with anterior plating and bilateral posterior sacroiliac screws.

A Pfannenstiel incision was made to access and anatomically reduce the bilateral superior and inferior pubic rami fractures, which were subsequently stabilized with plate fixation. Attention was then directed to the SIJ fixation. Under fluoroscopic guidance using lateral, inlet, and outlet views, the entry point for screw placement was identified on the lateral view, located at the posterior aspect of the ilium, just lateral to the sacral neural foramina at the S1 level. Through a small (approximately 1 cm) incision on the left side, a 2 mm guidewire was advanced percutaneously across the SIJ, traversing the sacral body and crossing into the contralateral (right) SIJ, exiting through the right-sided skin (Fig. 1B-D). Correct guidewire trajectory was meticulously confirmed on both inlet (to verify anteroposterior alignment) and outlet (to assess cranio-caudal alignment) views.

Drilling was performed over the guidewire from the left side using a 4 mm cannulated drill bit, and screw length was measured. A 6.5 mm diameter, 80 mm long cannulated, partially threaded screw was then inserted from the left side. Subsequently, a small (~1 cm) incision was created at the right-sided guidewire exit point, where drilling was repeated using the same drill bit. After measuring screw length, a second 6.5 mm diameter, 70 mm long cannulated, partially threaded screw was inserted from the right side. Bilateral SIJ fixation was thus achieved using a single trans-sacral guidewire inserted from the left to the right side.

Screw placement was performed through the S1 ilio-sacral corridor, which was selected based on preoperative CT confirmation of an adequate trans-sacral osseous pathway and absence of sacral dysmorphism. Single-level S1 fixation was chosen because stable anterior ring fixation had already been achieved, symmetric reduction of both SIJ was obtained, and sufficient biomechanical stability was expected without the need for supplemental S2 screws.

## Discussion

We present a modification of posterior pelvic fixation for bilateral SIJ distraction injuries using a single guidewire passed across both SIJs and exiting contralaterally. This technique provides a shared reference point for bilateral screw placement, enabling controlled screw trajectories and simultaneous compression across both joints. In this case, outlet imaging confirmed the guidewire was positioned cranial to the L5 neural foramina, and postoperative evaluation demonstrated intact L5 nerve function bilaterally.

Percutaneous ilio-sacral screw fixation remains a standard treatment for SIJ injuries but typically requires separate guidewire insertions for each side, increasing surgical time and radiation exposure^[10,11]^. Previous studies have demonstrated that tools such as coaxial guide systems improve efficiency by reducing fluoroscopy use^[12]^. Our single-wire bilateral approach achieves similar workflow advantages while offering the biomechanical benefits of trans-iliac–trans-sacral fixation, including greater screw purchase and improved load-sharing capacity^[13]^.

This technique’s main benefits include fewer wire passes, less fluoroscopy, and easier application of symmetric compression across both SIJs. However, safe use relies on meticulous preoperative CT planning and intraoperative imaging, particularly inlet and outlet views. In this case, outlet views alone were used for contralateral placement, consistent with prior reports showing acceptable outcomes despite a small risk of malposition^[14]^.

This technique is applicable primarily in acute, symmetrically reduced bilateral SIJ disruption patterns after restoration of anterior ring stability. It is not intended for chronic injuries, vertical sacral fractures, sacral dysmorphism, or cases requiring divergent screw trajectories. Although a single long trans-iliac–trans-sacral screw is a valid option when a continuous safe corridor exists, bilateral ilio-sacral screws were selected in this case to allow independent compression across each SIJ and to accommodate minor side-to-side variations in joint reduction. Previous biomechanical studies suggest that bilateral ilio-sacral fixation can provide greater rotational stiffness compared with a single long screw, particularly in bilateral injuries^[15]^.

Modern navigation and robotic systems have demonstrated improved accuracy and reduced radiation exposure during ilio-sacral screw placement; however, these technologies require specialized equipment and increased costs. Studies by Klingebiel et al. and Timmer et al. have shown favorable results with navigation-assisted techniques, particularly in complex anatomy. In contrast, the present technique relies solely on conventional fluoroscopy and may be especially relevant in resource-limited settings where navigation systems are unavailable.^[16,17]^

The primary limitation of this report is that it describes a single case without comparative data. This method may not be suitable for patients with sacral dysmorphism, narrow osseous corridors, or asymmetric reductions. Using the method described, the ilio-sacral screws cannot be inserted in a slight caudal to cranial direction which is perpendicular to SIJ and also posterior to anterior direction of screw cannot be maintained. Another limitation of this report is that some fluoroscopic images were captured from the operating room monitor rather than exported digitally, which may affect image resolution. The decision to use single-level fixation should be individualized and is therefore included as a procedural limitation. Further clinical research and biomechanical studies are needed to confirm its safety, define indications, and quantify potential reductions in operative time and radiation exposure.

## Conclusion

A single guidewire traversing both SIJ offers a straightforward technique that reduces operative time and radiation exposure while enabling effective bilateral screw fixation. It is suitable for both obese and nonobese patients, though it requires a long 2 mm guidewire. Precise use of fluoroscopic landmarks is essential to maintain neural safety.
